# L1 Grammatical Gender Variation through the Representation in the Lexicon

**DOI:** 10.1007/s10936-022-09867-7

**Published:** 2022-04-01

**Authors:** Rachel Klassen, Björn Lundquist, Marit Westergaard

**Affiliations:** 1 Department of Language and Culture, UiT The Arctic University of Norway, Tromsø, Norway; 2 Department of Language and Literature, Norwegian University of Science and Technology (NTNU), Trondheim, Norway

**Keywords:** Grammatical gender, L1 variation, Lexical access, Gender representation, Norwegian

## Abstract

In most studies on gender processing, native speakers of the same language are treated as a homogeneous group. The current study investigates to what extent an ongoing change in the gender system of Norwegian (a development from three to two genders, involving the loss of feminine) may be reflected in processing. We carried out a gender decision task in which speakers were presented with 32 nouns of each gender (masculine, feminine, neuter) and asked to select the corresponding indefinite article. Based on these results, we identified three different groups: three-gender speakers, two-gender speakers, and an unstable gender use group that used feminine gender to varying degrees. This division corresponded with clear differences in RTs, the two-gender speakers being faster overall with no difference across conditions, the three-gender group being slower with masculine, and the unstable group being slower with both masculine and feminine. Thus, our results indicate that native speakers of the same language can in fact have different underlying representations of gender in the lexicon.

## Introduction

Grammatical gender is an abstract feature inherent to the noun that is present in approximately 44% of all languages of the world (Corbett, 2013). According to the standard definitions of gender (Corbett, 1991; Hockett, 1958), the feature is only visible as agreement on other targets, such as determiners or adjectives. Furthermore, gender assignment is often non-transparent, with few (or no) links between the gender of a noun and both its meaning and in many cases the morphophonological shape of the noun. Given this complexity, grammatical gender has proven to be a fruitful area for research in multiple fields of linguistics. From a psycholinguistic perspective, such research has examined the representation and processing of gender in the lexicon across numerous child and adult populations and from both L1 and L2 perspectives (e.g. Brouwera et al., 2017; Dussias et al., 2013; Lemmerth & Hopp, 2019; Sagarra & Herschensohn, 2010). Representational studies examining a variety of different languages (for a review, see Klassen, 2016; Sá-Leite et al., 2019) have consistently treated native adult speakers of each language as a homogeneous group. This can be problematic, for the following reasons: (1) There are numerous linguistic contexts in which language variation clearly affects the use of grammatical gender, and (2) not allowing for the possibility that native speakers have different representations even in their L1(s) also runs counter to recent findings in bilingualism research regarding the impact of individual differences (e.g. Dörnyei, 2014).

In spite of this keen interest in grammatical gender across different populations and fields of linguistics, there are still many aspects of this feature that are under debate. By changing the perspective on L1 groups, we aim to elucidate the representation of gender systems in the context of language variation, contributing important novel evidence to the field of lexical access.

## Previous Research

### Representation of Gender in the Lexicon

Models of lexical access, including both spoken word production and visual word recognition, is an area that enters into the debate on grammatical gender. The level of representation of grammatical gender, the context in which gender information becomes available as well as the mechanisms by which it is selected is not only an area of disagreement across models, but in some cases is merely discussed as a side note.

Language production models such as *WEAVER*++ (Levelt, 1993; Levelt et al., 1999; Roelofs, 1992, 1997), the *interactive two-step model* (Dell et al., 1997) and the *interactive activation model* (McClelland & Rumelhart, 1981; Rumelhart & McClelland, 1982) of word recognition posit that grammatical gender is represented as a feature of the lemma, or syntactic word, which is accessed prior to morphonological information in the case of models such as *WEAVER*++ that assume discrete processing. In contrast, in the *independent network model* of language production (Caramazza, 1997), gender information belongs to a syntactic feature network that is independent of the networks storing lexical-semantic and morphophonological information.

**Fig. 1:**
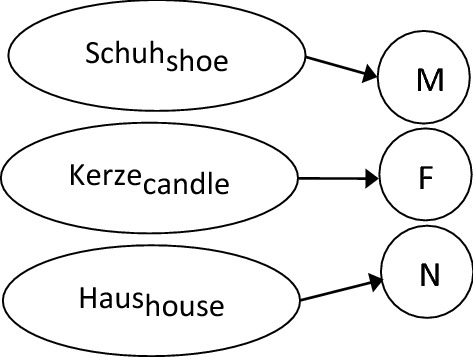
Representation of the German gender system in the lexicon

Regardless of the level of the feature, most models seem to converge on the notion that gender information is represented as a set of nodes (or determiner forms in the case of the *independent network model*) to which the relevant lexical entries are linked. An example of the representation for German is shown in Fig. 1.

### L1 Lexical Access Research

Evidence regarding the representation of grammatical gender in the mental lexicon was first offered by Schriefers (1993) for L1 Dutch speakers. In this study, native Dutch speakers performed two L1 picture-word interference tasks in which they were asked to name the picture while ignoring the written distractor word. The stimuli consisted of pictures and words with either the same gender (gender-congruent condition) or a different gender (gender-incongruent condition) and the results showed significantly faster reaction times (RTs) for gender-congruent picture-distractor pairs than for gender-incongruent ones, an effect which Schriefers labelled *the gender congruency effect*.

Since this initial study, the effect of gender congruency has been investigated using the picture-word interference paradigm in numerous L1 studies, with the findings diverging even within the same language groups (Table 1). We are not aware of any previous studies that have investigated the effect of L1 grammatical gender variation on the representation in the lexicon, despite the relevance of considering L1 individual differences. In the present study, we investigate the representation and processing of grammatical gender in Norwegian by L1 Norwegian speakers. Norwegian is an ideal linguistic context for such a study, given the diversity among the many dialects spoken in Norway as well as the ongoing change to the Norwegian gender system that has recently been attested. Specifically, we ask (i) how L1 dialectal variation affects the representation of grammatical gender in the lexicon; and (ii) whether there are fundamentally different representations of L1 grammatical gender, and if so, how these representations are borne out in language processing. In the remainder of the paper, we offer a summary of gender in Norwegian ("Gender in Norwegian" section), details on the participants and design of the present study ("The present study" section), the results ("Results" section), a discussion ("Discussion" section) and conclusions ("Conclusion" section).

**Table 1:** Summary of findings from L1 gender studies

Language	Gender congruency effect?	Studies
Italian	Yes	Cubelli et al. (2005), Paolieri et al. (2010), Paolieri et al. (2011)
	No	Miozzo and Caramazza (1999), Cubelli et al. (2005)
French	Yes	Alario et al. (2008)
	No	Alario and Caramazza (2002)
Catalan	No	Costa et al. (1999)
Spanish	Yes	Alario et al. (2008), Paolieri et al. (2010)
	No	Costa et al. (1999)
Dutch	Yes	Schriefers (1993), Schiller and Caramazza (2003), van Berkum (1997), La Heij et al. (1998), Starreveld and La Heij (2004)
	No	Schiller and Caramazza (2003), van Berkum (1997), La Heij et al. (1998), Starreveld and La Heij (2004)
German	Yes	Schriefers and Teruel (2000), Schiller and Caramazza (2003), Alario et al. (2008)
	No	Schiller and Caramazza (2003)
Greek	Yes	Plemmenou et al. (2002)

## Gender in Norwegian

### The Norwegian Gender System

While some Germanic languages, notably Dutch, Swedish and Danish, have undergone a historical change from a tripartite system to a binary one (common and neuter), Norwegian dialects have generally retained masculine, feminine and neuter.^1^ The masculine and feminine gender paradigms are very similar, and this syncretism has only increased over the last 400–500 years, while the neuter paradigm has kept or developed unique exponents. The traditional three-gender system of most varieties of spoken Norwegian is illustrated in Table 2. Gender is marked on the indefinite article (*en, ei* or *et*, for masculine, feminine or neuter respectively) and the definite suffix, as well as adjectives in the strong declension (indefinite), possessives, and the prenominal determiner in double definite forms (required with modified definite nouns).

**Table 2:** The traditional three-gender system in many varieties of spoken Norwegian

	Masculine	Feminine	Neuter
Indefinite	**en** stol *a chair*	**ei** bok *a book*	**et** hus *a house*
Definite	stol**en** *chair.def*	bok**a** *book.def*	hus**et** *house.def*
Double definite	**Den** røde stol**en** *The red chair.def*	**den** røde bok**a** *the red book.def*	**det** røde hus**et** *the red house.def*
Adjective	en **fin** stol *a nice chair*	ei **fin** bok *a nice book*	et **fint** hus *a nice house*
Possessive	**min** stol/stol**en min** *my chair*	**mi** bok/bok**a mi** *my book*	**mitt** hus/hus**et mitt** *my house*

Due to the extensive syncretism between the masculine and the feminine gender shown in Table 2, the difference between the masculine and the feminine is only visible in two forms: the indefinite article and the possessive. Neuter has its own forms for strong adjectives and prenominal definite articles in addition to the possessive and indefinite article form.

With respect to written Norwegian, there are two standards: *bokmål* and *nynorsk*. While *nynorsk* (based on the dialects) requires the use of all three genders for the indefinite article and the possessive, *bokmål* (based on Danish) may be used with either a three- or a two-gender system.^2^*Bokmål* is by far the more widely used written standard, as most books, magazines, newspapers and subtitles are written in this variety (Lundquist & Vangsnes, 2018; Vangsnes et al., 2017), and only 12.2% of all school children in Norway have *nynorsk* as their main written language (Vangsnes et al., 2017). In most *bokmål* media and books, the feminine exponents are rarely used—especially not as indefinite articles—meaning that dialect speakers are constantly exposed to a two-gender system.

In recent years, changes in the gender system have been reported in a number of dialects. Based on a spoken corpus of 142 speakers in Oslo, Lødrup (2011) shows that the feminine indefinite article is used infrequently among older speakers and is virtually non-existent in the production of the younger generation. A similar change is attested in two other major cities, Tromsø and Trondheim (Busterud et al., 2019; Rodina & Westergaard, 2015). In all these instances, the feminine indefinite article *ei* is replaced by masculine *en*, while the definite suffix *-a* is retained in its original form; thus, the emerging two-gender system has the pattern ***en**** bok-bok****a*** ‘a book-the book’ for previously feminine nouns.

It is relevant in the context of this study to note that gender assignment in Norwegian is generally non-transparent. According to Trosterud (2001), who has based his calculations on 31,500 nouns in the *Nynorsk Dictionary*, masculine nouns make up 52%, feminine nouns 32% and neuter nouns only 16%. By Rodina and Westergaard’s (2015) count in a corpus of child-directed speech (adult Tromsø speakers with a three-gender system), the token frequency of masculine nouns in the input is even higher—62.6%—while feminine and neuter nouns are at 18.9% and 18.5%, respectively. This means that in a two-gender system, the common gender (resulting from the convergence of masculine and feminine) will be significantly more frequent than the neuter, at around 80% (similar to Dutch).

### Gender Processing in Norwegian

In a series of studies, Lundquist and colleagues (Lundquist & Vangsnes, 2018; Lundquist et al., 2016) examined whether dialectal differences between Norwegian spoken in the Northern, Western, and Eastern regions affect adult L1 speakers’ predictive use of gender in auditory processing. The results showed that gender prediction did vary across dialects, and that speakers from these regions differed significantly in their predictive use (or lack thereof) of masculine and feminine. L1 speakers from Western Norway (*nynorsk* area) consistently used gender predictively for masculine, feminine, and neuter nouns. In contrast, in Northern Norway (*bokmål* area), even speakers who used three genders in production only used neuter in predictive processing. Those speakers in the North who only produced two genders (masculine and neuter) used both of them in prediction, unlike speakers from Eastern Norway who also had a binary gender system though only used neuter predictively. Together, these findings clearly show that L1 speakers of different Norwegian dialects use gender differently in processing, and suggest that such variation may also affect the representation of gender information in the lexicon of L1 Norwegian speakers. The present study explores the gender representation that underlies these effects seen in predictive processing.

## The Present Study

### Participants

A total of 239 L1 Norwegian adults from across Norway participated in this study (M age = 39 (range 15–75), SD = 15; 88 males, 151 females). Participants were recruited from across Norway, as indicated by the geographical region of the city or town of their self-reported dialect: Eastern Norway (N = 66), Western Norway (N = 37), Mid-Norway (N = 31) and Northern Norway (N = 76). While it was not possible to recruit an even regional distribution of speakers due to the constraints of web-based data collection, importantly, the results we obtain are in accordance with previously found correlations between gender systems, age and dialects.^3^ Ninety-nine participants (41%) reported intermediate proficiency in a language with grammatical gender that learned after the age of 5 [German (N = 45), French (N = 21), Spanish (N = 18) or other gendered languages (N = 15)]. In order to mitigate any possible cross-linguistic influence resulting from additional language knowledge on the data, an accuracy threshold was established (further described in "Materials and Response accuracy" sections).

### Method

The participants performed a gender decision task in which they were presented with bare nouns in Norwegian and asked to select the corresponding indefinite article (*en*_*M*_, *ei*_*F*_ or *et*_*N*_) as quickly and as accurately as possible. Importantly, the instructions emphasized to the speakers that there were no correct answers and that they should answer according to their own dialect. In order to train the participants on the task and the use of three response keys (**N** for *en*, **I** for *ei*, and **T** for *et*), a short practice session preceded the experimental stimuli. Throughout the task, each noun (randomized by the software) appeared on the screen until the participant responded, or for a maximum of 3500 ms. The entire experiment (including a short language background questionnaire) took approximately 5 min to complete.

Data collection was conducted via Ibex Farm (spellout.net/ibexfarm), a web platform that hosts online experiments in a browser. For each stimulus, Ibex Farm recorded the response key pushed as well as the reaction time (RT). Participants were recruited through social media or email and completed the experiment at home using their private computers, allowing for a much wider recruitment of participants. While not a conventional lab setting, the possible variation in the data resulting from the use of different computers was minimized through the design of the platform itself (i.e., the entire experiment is loaded on the individual computer prior to the start of the experiment such that RT differences between experimental conditions are independent of computer processing and internet connection speeds) and by recruiting a substantial number of participants.

### Materials

The task consisted of 108 Norwegian nouns, 12 practice and 96 experimental stimuli (see “Appendix A”). These inanimate, count nouns were divided equally into conditions based on the traditional gender of the noun (i.e., 36 masculine, 36 feminine and 36 neuter). The stimuli were reviewed by two native speakers of Norwegian, one from Northern Norway (*bokmål* area) and one from Western Norway (*nynorsk* area) in order to avoid specific nouns that varied in either form or gender across different dialects.^4^ There were no significant differences across gender conditions in mean number of letters (p = 0.354), number of syllables (*p* = 0.560), or mean word frequency by web (*p* = 0.998; TenTen, Jakubíček et al., 2013) or subtitle measures (*p* = 0.311; Opus2, Tiedemann, 2012).

## Results

### Response Accuracy

Overall, the participants chose the correct gender (defined as the traditional gender of the noun) in 85% of the trials. As regards the three gender conditions, participants performed at ceiling for masculine (masc; 95%) and neuter (neut; 96%), while—unsurprisingly in light of the language changes reported in section the Norwegian gender system—they showed more variation in the feminine condition (fem; 63%). A very small subset of the participants scored below 80% on the masculine and neuter conditions (N = 12) and were not included in the analyses, as this suggested that they were either significantly affected by cross-linguistic influence from proficiency in additional languages or that they did not use the response buttons according to the task instructions.

Responses other than the feminine indefinite determiner (*ei*) for nouns in the fem condition were not evenly distributed among the participants: some participants never used *ei*_F_, others used it consistently, while others still used it to varying degrees. Given that both previous research as well as the current dataset show that feminine is the locus of variation in Norwegian gender, participants were divided into groups based on their responses in the fem condition. Participants who used *ei*_F_ with 0–2 feminine nouns (maximum of 6%)^5^ were classified as the two-gender group (N = 46)^6^; participants who used *ei*_F_ with 29–31 feminine nouns (minimum of 94%) were put into the three-gender group (N = 92); and all participants who displayed more variation in their use of *ei*_F_ (3–28 nouns, 10–90%) formed the unstable gender group (N = 89).

**Fig. 2:**
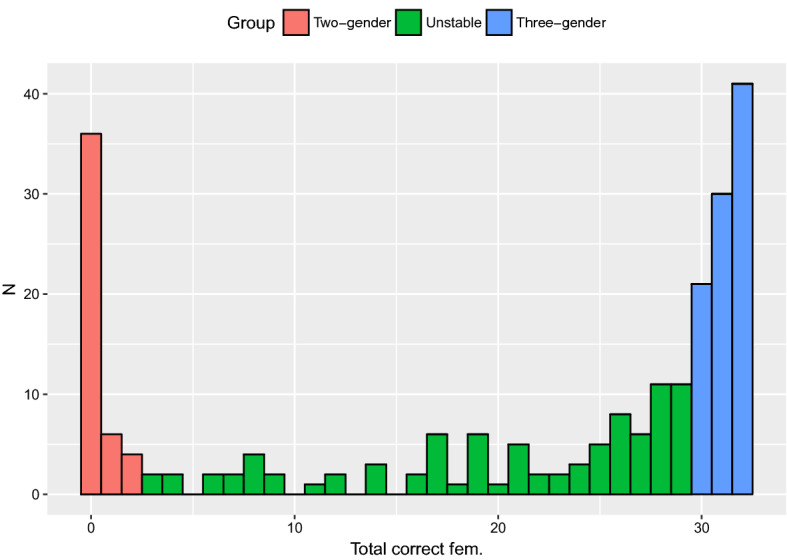
Participant groups according to the number of feminine determiner responses

In light of the ongoing changes in the Norwegian gender system (Sect. the Norwegian gender system), it is probable that these groups reflect a representative sample of Norwegian gender use. That is, the largest group of speakers still has a stable three-gender system, significantly fewer speakers have already developed a stable two-gender system, and another large group has an unstable system as it transitions from three to two-genders. Figure 2 illustrates these three speaker groups, showing the distribution of participants according to the number of *ei*_F_ responses. As can be seen in the figure, there is considerable variation within the unstable group: some of the speakers seem to have an almost intact three-gender system, while others rarely use the feminine at all. Still, we choose to treat this as one group characterized by a competition between feminine and masculine exponents for the traditionally feminine nouns. Note that it is always the masculine *en* determiner (rather than neuter *et*) that is used in the fem condition when feminine *ei* is not selected.

### Reaction Times

To analyze the RT data, we fit a series of mixed effects models to estimate the effects of Group (two-gender, three-gender, unstable) and Condition (masc, fem, neut), as well as the interaction between Group and Condition, using the lme4 package in R (Bates et al., 2015). Our dependent measure is the log-transformed RTs and the models include random intercepts for participant and stimulus, as well as a by-participant slope for Condition. We obtain *p*-values for the main effects and interactions from a series of likelihood ratio tests (using the anova function in lme4) and from pairwise comparisons between groups and conditions using the emmeans package (Lenth, 2021). The full regression table is given in “Appendix B”.

**Fig. 3:**
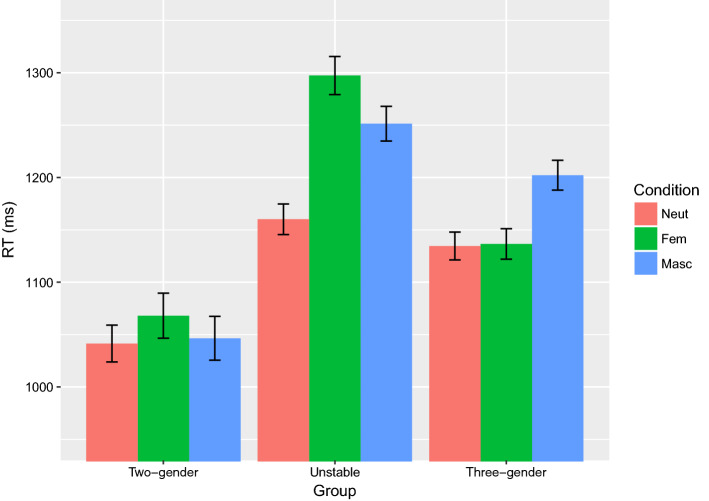
RTs in each condition by group. Error bars represent 95% confidence intervals

Results show a main effect of Condition (χ^2^(2) = 9.6, p < 0.01) and Group (χ^2^(2) = 25.5, *p* < 0.001), as well as an interaction between Condition and Group (χ^2^(4) = 67.4, *p* < 0.001). With respect to Condition, neut is overall faster than both fem and masc (both *p*s < 0.01), with no significant difference between fem and masc. In terms of Group, the two-gender group is overall faster than the unstable and three-gender groups (both *p*s < 0.001), and there is no significant difference between the unstable and the three-gender groups. RT results broken down by group are shown in Fig. 3.

To determine the locus of the interactions for Group and Condition, we consider the three participant groups separately and do pairwise comparisons for the three gender conditions for each group (values extracted by the emmeans package from the full model specified above, statistics for all the pairwise comparisons within each group given in “Appendix C”). For the two-gender group, there is no significant difference in RTs by condition. Data from this group illustrate that none of the conditions are inherently more difficult, and thus the differences across conditions in the unstable and three-gender groups are presumably not triggered by general problems of lexical access. In the unstable group, the neut condition is faster than the other two conditions (both *p*s < 0.001), with no significant difference between fem and masc. Finally, in the three-gender group, the masc condition is significantly slower than fem and neut (both *p*s < 0.001), while we see no difference between fem and neut.

### Frequency-Driven Effects

Having established that there is significant variation among the participants with respect to the fem condition—both in terms of the number of *ei*_F_ responses ("response accuracy" section) and RTs ("reaction times" section)—we now examine how the frequency of feminine exponents in the input may have affected the responses and RTs in the fem condition.^7^ To this end, the frequencies of the lemma (e.g., *bok*, ‘book’), the definite feminine suffix -*a* (e.g., *bok****a***, ‘the book’) and the feminine indefinite determiner *ei*_F_ (e.g., ***ei**** bok*, ‘a book’) for each of the feminine noun stimuli are considered in "frequency-driven effects" section. Frequencies are based on the NoWaC corpus (Guevara, 2010).^8^

#### Frequency Effects on the Choice of ***ei***_F_

To start, we examine the relationship between input frequency and participants’ use of the feminine determiner with traditionally feminine nouns. We focus exclusively on the unstable group, as there was little to no variation in the response data for the two-gender and three-gender groups.

**Fig. 4:**
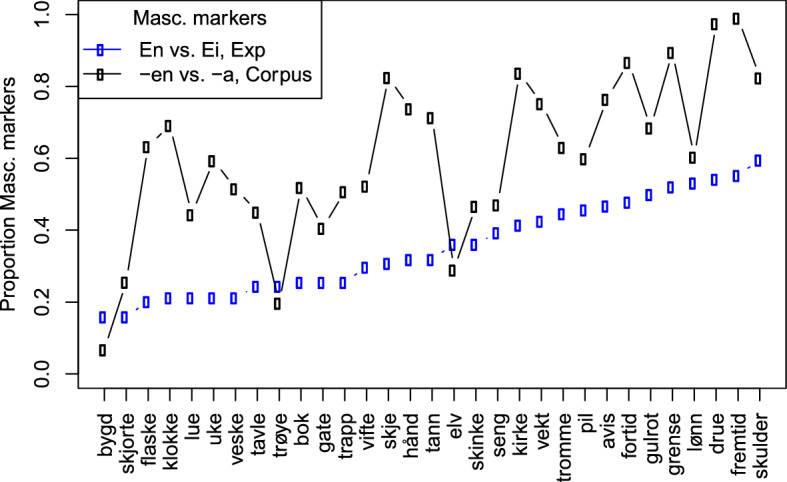
The proportion of *en* vs. *ei* responses in the experiment and *-en* vs. *-a* suffixed forms in the corpus data

The rate at which the unstable group used *ei*_F_ in the fem condition varied considerably according to the individual stimuli, from 40% (*skulder*, ‘shoulder’) to 84% (*bygd*, ‘village’). With respect to the different frequency measures acquired from the NoWaC corpus, we find no effect of the absolute feminine noun frequency by lemma (e.g., *bok*), definite form (e.g., *bok****a****/bok****en***), or indefinite form (e.g., ***ei/en**** bok*) (all *p*s > 0.5). Rather, the best predictor for the unstable group’s use of *ei*_F_ is the proportion of definite feminine forms (e.g., *bok****-a***_***F***_ vs. *bok****-en***_***M***_) with which each stimulus appears in the written corpus (β = − 2.38, se = 0.49, z = − 4.87, *p* < 0.001). That is, the more often a feminine noun appears with the masculine definite suffix *-en*, the more likely the participants are to use the masculine indefinite determiner *en* in the experiment. The relation between *en*_*M*_/*ei*_*F*_ responses in the fem condition in the experiment and the proportion of masculine definite endings in the corpus are shown in Fig. 4, with all the fem condition stimuli listed along the X axis by lowest to highest proportion of *en* vs. *ei* responses.^9^

#### Effect of masc/fem Proportions on RTs

In this section, we consider whether the proportion of the masculine definite *-en* suffix affects RT results in the two-gender, three-gender and unstable groups differently. Comparing the effects of the absolute frequencies (lemmas, definite form, indefinite form), the frequency of the unambiguous feminine forms (*ei*, suffix -*a*), and the proportion of masculine vs. feminine forms (e.g., ***en**** bok* + *bok****en*** vs. ***ei**** bok* + *bok****a***), we find no effect of the absolute frequency measures on RTs, and only small effects of the frequency of unambiguous feminine forms, either as main effects or interactions with Group.

**Fig. 5:**
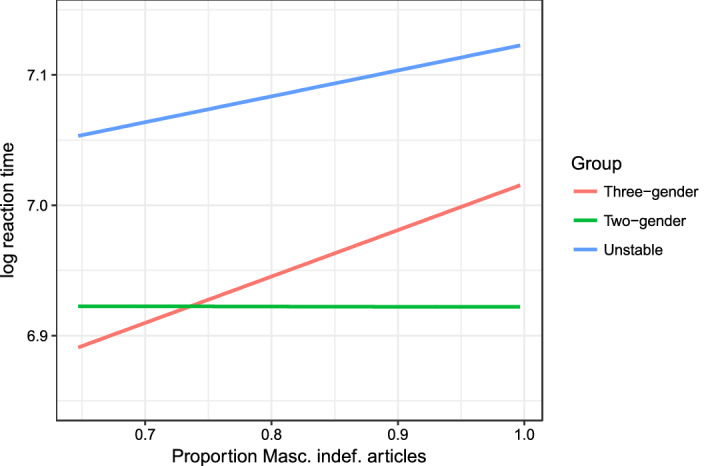
The relation between the log RTs and the proportion of *en*_*M*_ vs. *ei*_*F*_ used with traditionally fem nouns in the corpus

As regards the RTs, the best predictor is the proportion of masculine vs. feminine forms. We only focus on the indefinite article, i.e., the proportion of the masculine indefinite article *en* for the feminine nouns. We find that the effect of the indefinite determiner only emerges as an interaction with Group (χ^2^(2) = 10.57, *p* < 0.01, statistics from likelihood ratio tests, with random intercepts for Participant and Item, and in addition total frequency (log-transformed) as a fixed effect). The three-gender group responds more slowly to feminine nouns that frequently appear with the masculine determiner (β = 0.38, se = 0.18, t = 2.077), while the two-gender group is unaffected by the proportion of *en*_*M*_ vs. *ei*_*F*_. This interaction is illustrated in Fig. 5, see “Appendix D” for the full regression table.

Based on the number of *ei*_*F*_ responses it seems that the three-gender group has a stable tripartite gender system, though they are in fact the group whose RTs are most highly affected by the proportion of masculine vs. feminine determiners in their input. These additional insights from the RT data seem to suggest that the three-gender group’s system is not as stable as their use of the feminine form *ei*_*F*_ indicates.

The unstable group’s RTs are also affected by the proportion of *en*_*M*_ vs. *ei*_*F*_, though surprisingly to a lesser extent than the three-gender group (again, see “Appendix D” for model estimates). As we saw in "frequency effects on the choice of ei_F_" section, the unstable group’s number of *ei*_*F*_ responses is further affected by the proportion of *-en* vs. *-a* definite suffixes in the input, and thus it seems as though the unstable group is aptly named in that they do indeed have an unstable gender system.

In contrast to the other groups, the two-gender group is unaffected by the variation between masculine and feminine forms (neither indefinite determiners nor definite suffixes) in the input.

#### Frequency Effects on Masculine and Neuter Nouns

We have established that the three groups differ in their response patterns and their RTs, with certain frequency measures for the nouns in the fem condition affecting response patterns to a greater or lesser extent in each group. We now turn our attention to possible frequency effects on the stimuli in the masc and neut conditions, as well as possible interactions with Group. To test this, we compare models with a number of predictors (Condition, Group, and the three frequency measures described in "effect of masc/fem proportions on RTs" section). We find no interaction between Group and Frequency (by any measure), nor any interaction between Condition and Frequency. When comparing the different frequency values (lemma, definite forms, indefinite forms) we only find an effect of the frequency of the indefinite article (χ^2^ = 6.3, df = 1, *p* < 0.05), which is not unexpected given that the task asked participants to choose an indefinite determiner form.^10^

## Discussion

The data from the gender decision task show that L1 Norwegian speakers are generally consistent in their use of the masculine determiner with masculine nouns and the neuter determiner with neuter nouns (rates of 96% and 97%, respectively). Furthermore, their RTs are unaffected by the frequency of gender-marked forms in their input,^11^ suggesting that masculine and neuter have a stable representation in the gender system of all native speakers of Norwegian. This is in stark contrast to the use of the feminine determiner with traditionally feminine nouns, where the speakers show three distinct patterns: no use of feminine (two-gender group), consistent use of feminine (three-gender group), or inconsistent use of feminine (unstable group). As the focus of the present study is gender variation in L1 speakers, in the remainder of the discussion we focus on the data for feminine nouns, addressing each speaker group in turn.

### Three-Gender Group

The response data of the three-gender group show consistent use of the feminine determiner (with a maximum of 6% variation; Fig. 2). From a language use perspective, it is clear that these speakers have a gender system clearly consisting of masculine, feminine and neuter. The RT data, however, suggest that the underlying representation of this tripartite gender system may not be as stable as the language use indicates, as these speakers were the most affected by variation in masculine vs. feminine determiners in the input (Fig. 5). The RTs for this group further show that responses for masculine nouns are significantly slower than for both feminine and neuter nouns (Fig. 3). These are not the first findings pointing to longer RTs for masculine nouns in a language a with tripartite gender system: Opitz and Pechmann (2016) report a series of online experiments with L1 German speakers who consistently display longer RTs for masculine nouns in German. These authors offer a theoretical account involving underspecification of gender features which is beyond the scope of the current study, and we are unaware of any existing psycholinguistic explanation for the apparent processing costs borne by masculine nouns. Though the design of our current study does not allow us to propose such an account, this is relevant avenue for future research as findings in this area will undoubtedly further our understanding of gender in lexical access.

**Fig. 6:**
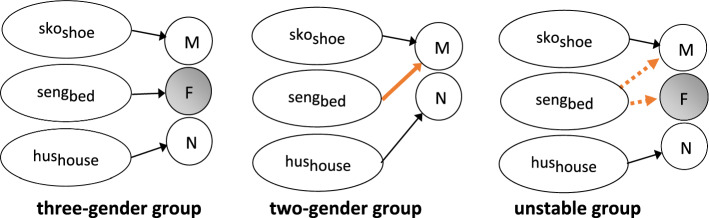
Representations of the L1 Norwegian gender system in the lexicon across the three speaker groups

From a representational perspective, it seems as though this group of Norwegian speakers have a gender representation much like that shown in Fig. 1 for German. In the lexicon, masculine nouns are linked to the masculine gender node, feminine nouns to the feminine node, and neuter nouns to the neuter gender node. The links from the masculine and neuter nouns to their respective nodes are stable, while the interaction found between input frequencies and RTs suggest that the links between the feminine nouns and the feminine node are destabilizing (Fig. 6). Furthermore, if this destabilization process continues on the same trajectory, it is likely that three-gender group speakers would join the unstable group, and eventually also the two-gender group.

### Two-Gender Group

Like the three-gender group, the two-gender group is also consistent in their gender use, though in this case they do not use the feminine determiner (with a maximum of 6% variation; Fig. 2). In contrast to the three-gender group, however, RT data support the notion that these speakers have a stable gender system, as they are unaffected by the masculine/feminine variation in their input (Fig. 5). They also display a different pattern of RT results than the previous group, with mean RTs for nouns of each gender being statistically equal.

These results suggest that this group of speakers has a stable bipartite representation of gender in Norwegian. This two-gender representation has links between the masculine and neuter nouns and their respective nodes; however, in this case the traditionally feminine nouns are linked to the masculine gender node. This link between feminine nouns and the masculine node appears to be well-established and stable (Fig. 6).

### Unstable Gender Group

The unstable gender group is characterized by a wide variation in their use of the feminine determiner (between 10 and 90%; Fig. 2). Not only was their feminine use inconsistent, but it was also significantly affected by the variation between the masculine definite suffix *-en* and the feminine definite suffix *-a* in the input (Fig. 4). In a similar manner, this group’s RTs were affected by the variation between the masculine and feminine indefinite determiners (Fig. 5). They further differed from both of the other two groups in the pattern of their RTs: responses for neuter were significantly faster than both masculine and feminine nouns, which did not differ statistically. This arguably reflects the fact that neuter is the only representation unaffected by the instability of their gender system. While the use of masculine is stable, its representation is affected by the increasing use of masculine exponents with traditionally feminine nouns.

Given that speakers in this group do use feminine gender, it seems that they still do have a representation for feminine. However, considerable instability comes from the fact that feminine nouns are becoming increasingly linked to the masculine gender node, as these speakers’ systems move towards the complete loss of feminine (as in the two-gender group). The variation in the use of feminine exponents with each noun as seen in the corpus data (Fig. 4), together with the variation in feminine use across speakers in this group, likely means that the degree to which nouns are linked to the feminine and/or masculine nodes vary both in terms of the specific lexical item as well as across speakers. In some cases, links between traditionally feminine nouns and the feminine node would be the strongest, in other cases, links to the masculine node would be stronger, and in others still the links would be approximately equal as specific nouns and speakers move through this process of change in gender in Norwegian. The inconsistent nature of these links with traditionally feminine nouns results in processing delays due to increased competition between the masculine and feminine gender nodes that must be resolved prior to the selection of gender information.

### Representation of L1 Gender Variation in the Lexicon

In light of the unique RT patterns for each of the speaker groups (Fig. 3), we have proposed three different representations of the Norwegian gender system which are illustrated in Fig. 6.

We therefore submit that it is indeed possible for different L1 speakers to have different underlying representations of grammatical gender in the lexicon in L1 variation contexts. Furthermore, these representations affect language processing differently: for speakers with a tripartite gender system, responses for masculine nouns show the highest processing costs; those with an unstable gender system display processing costs with both masculine and feminine gender; and speakers who have a binary system show no significant processing costs by gender at all. The results from the unstable gender group in particular leave questions open for further research, including the extent to which the instability in the gender system is due to specific lexical items (i.e., if speakers consistently use masculine or feminine determiners depending on the noun) and whether their behaviour would differ in gender assignment vs. gender agreement contexts.^12^

These findings have important implications beyond Norwegian, and will hopefully propel forward further work on the representation of gender information in language variation. There are many contexts in which gender variation has been documented but not examined with respect to the implications for lexical access (e.g. Danish and Swedish, Josefsson, 2014). In a few cases, studies have offered important evidence regarding gender in child bidialectal acquisition (e.g. Venetian and Italian: Kupisch & Klaschik, 2017; Dutch and dialects of Dutch in the Netherlands: Cornips & Hulk, 2008), leaving the representational perspective as a fruitful avenue for future work.

Another important contribution of the present study regards the traditional assumption in linguistic research that all speakers with a similar linguistic profile form a homogeneous group. Our results clearly show that speakers with the same L1 should not necessarily be treated as a single group, though this is perhaps less surprising given that it is well-known that there is significant gender variation in Norwegian. It is, however, also relevant to note that individual differences between speakers are increasingly shown to be important, particularly in the context of bilinguals (e.g. Bayram et al., 2017; Dörnyei, 2014; Flores & Rinke, 2020), though this is arguably also relevant for L1 speakers (e.g. Dąbrowska, 2012). Outside of sociolinguistic factors such as socioeconomic status and education level as well as popular psycholinguistic measures like working memory capacity, individual differences are not often considered at the level of language representation in lexical access. In this same vein, future psycholinguistic studies should consider speakers’ gender representations on an individual basis, along the lines of the gender use strategy analyses in both child and adult offline code-switching data (e.g. Cantone & Müller, 2005; Klassen, 2016).

## Conclusion

This study has offered novel evidence for representational differences in the lexicon of L1 speakers of Norwegian, showing not only that speakers with the same L1 can have different underlying representations for grammatical gender, but also that these representations have different consequences in language processing. To the best of our knowledge, this is the first study to examine the impact of gender variation on lexical access in the L1, and the results suggest that this should be an important area for further research, investigating other contexts of language variation, as well as different age groups.

## Appendix A: Stimuli

**Table:**

Noun	Gender	Stimulus type	Translation
blomkål	M	Practice	Cauliflower
dessert	M	Practice	Dessert
fordel	M	Practice	Advantage
høst	M	Practice	Autumn
bukse	F	Practice	Pants
pute	F	Practice	Pillow
reise	F	Practice	Trip
stekepanne	F	Practice	Frying pan
hjerte	N	Practice	Heart
navn	N	Practice	Name
skjerf	N	Practice	Scarf
yrke	N	Practice	Profession
arm	M	Experimental	Arm
butikk	M	Experimental	Store
børste	M	Experimental	Brush
dal	M	Experimental	Valley
farge	M	Experimental	Colour
flyplass	M	Experimental	Airport
frakk	M	Experimental	Coat
frokost	M	Experimental	Breakfast
hatt	M	Experimental	Hat
innsjø	M	Experimental	Lake
kniv	M	Experimental	Knife
koffert	M	Experimental	Suitcase
krig	M	Experimental	War
løk	M	Experimental	Onion
munn	M	Experimental	Mouth
måne	M	Experimental	Moon
måned	M	Experimental	Month
nøkkel	M	Experimental	Key
ost	M	Experimental	Cheese
ring	M	Experimental	Ring
rygg	M	Experimental	Back
ryggsekk	M	Experimental	Backpack
sko	M	Experimental	Shoe
skriver	M	Experimental	Slice
skygge	M	Experimental	Shadow
sommer	M	Experimental	Summer
stein	M	Experimental	Rock
stemme	M	Experimental	Voice
stol	M	Experimental	Chair
størrelse	M	Experimental	Size
sykkel	M	Experimental	Bicycle
vegg	M	Experimental	Wall
avis	F	Experimental	Newspaper
bok	F	Experimental	Book
bygd	F	Experimental	Village
drue	F	Experimental	Grape
elv	F	Experimental	River
flaske	F	Experimental	Bottle
fortid	F	Experimental	Past
fremtid	F	Experimental	Future
gate	F	Experimental	Street
grense	F	Experimental	Border
gulrot	F	Experimental	Carrot
hånd	F	Experimental	Hand
kirke	F	Experimental	Church
klokke	F	Experimental	Clock
leppe	F	Experimental	Lip
lue	F	Experimental	Beanie
lønn	F	Experimental	Salary
pil	F	Experimental	Arrow
seng	F	Experimental	Bed
skinke	F	Experimental	Ham
skje	F	Experimental	Spoon
skjorte	F	Experimental	Shirt
skulder	F	Experimental	Shoulder
tann	F	Experimental	Tooth
tavle	F	Experimental	Board
trapp	F	Experimental	Step
tromme	F	Experimental	Drum
trøye	F	Experimental	Jersey
uke	F	Experimental	Week
vekt	F	Experimental	Weight
veske	F	Experimental	Purse
vifte	F	Experimental	Fan
basseng	N	Experimental	Pool
belte	N	Experimental	Belt
besøk	N	Experimental	Visit
blad	N	Experimental	Leaf
bord	N	Experimental	Table
bryllup	N	Experimental	Wedding
egg	N	Experimental	Egg
fengsel	N	Experimental	Prison
flagg	N	Experimental	Flag
forsøk	N	Experimental	Attempt
gulv	N	Experimental	Floor
hjørne	N	Experimental	Corner
hode	N	Experimental	Head
hår	N	Experimental	Hair
jordbær	N	Experimental	Strawberry
kjøkken	N	Experimental	Kitchen
kjøleskap	N	Experimental	Refrigerator
klesskap	N	Experimental	Wardrobe
kne	N	Experimental	Knee
kontor	N	Experimental	Office
kyss	N	Experimental	Kiss
skjørt	N	Experimental	Skirt
skrivebord	N	experimental	Desk
slips	N	Experimental	Tie
tastatur	N	Experimental	Keyboard
tog	N	Experimental	Train
tre	N	Experimental	Tree
vindu	N	Experimental	Window
ønske	N	Experimental	Desire
øre	N	Experimental	Ear
øyeblikk	N	Experimental	Moment
år	N	Experimental	Year

## Appendix B: Model for Reaction Time

The predictors Condition and Group are dummy coded, with Feminine, Three-gender Group as intercept.

## Appendix C: Pairwise Comparisons within Each Gender Group

Pairwise comparisons within each gender group, obtained from the emmeans package (Lenth, 2021). Original model and emmeans commans given in addition.

## Appendix D: Model for Reaction Times for Feminine Nouns

Model for reactions times for the feminine nouns, as a function of Group (dummy coded, three-gender group as intercept), Proportion of masculine article (centered at mean), total frequency (log-transformed) and the interaction between Group and Proportion of masc. article as predictors.

## Footnotes

1 A notable exception is the Bergen dialect, which lost the feminine gender several hundred years ago, arguably due to language contact with Low German during the Hansa period (e.g. Jahr, 2001).

2 See e.g. Venås (1993) and Vikør (1995) for detailed information about the language situation in Norway.

3 That is, speakers from *nynorsk* areas use feminine gender more often than speakers from *bokmål* areas, and in the *bokmål* areas, older speakers use feminine much more than younger speakers (see "Gender in Norwegian" section and Authors, Forthcoming).

4 In spite of these control measures, in the analysis one noun of each gender (3 nouns total) had to be excluded due to unstable gender behaviour or deviant frequencies in the subsequent corpus analyses.

5 We allowed for a maximum of 2 accidental keystrokes in all groups.

6 That is, their gender systems appear to consist of only masculine and neuter.

7 While the stimuli were controlled for lemma frequency in both web and subtitle measures ("materials" section), given that gender is only visible on agreement targets and that Norwegians experience a lot of dialectal variation in their lives, it is relevant to consider how different frequency measures may interact with the trends in the data.

8 NoWaC is a web-based corpus with over 700 million words consisting primarily of texts from blogs and chat forums.

9 While the variation in the definite form is clearly the best predictor of the variation in the data, there is also a small effect of the (log) frequency of the indefinite feminine determiner *ei* on the response data (β = 0.128, se = 0.06235, z = 2.050, *p* < 0.05). This is not unexpected since the explicit instructions of the task were to choose an indefinite determiner form.

10 Note that this effect is independent of the non-significant effect of the definite form frequency measure.

11 With the exception of the frequency of the indefinite determiner forms *en*_*M*_ and *et*_*N*_, which are arguably task effects.

12 Thank you to an anonymous reviewer for pointing out these possibilities for future research.
